# Hyperplastic Polyposis and the smoking paradox in females

**DOI:** 10.1186/1897-4287-9-S1-P3

**Published:** 2011-03-10

**Authors:** Daniel D Buchanan, Mark A Jenkins, Aung K Win, Michael D Walsh, Diane M McKeone, Finlay Macrae, Christophe Rosty, Neal I Walker, Susan Parry, Joanne P Young

**Affiliations:** 1 Familial Cancer Laboratory, QIMR, Herston Q 4006, Australia; 2 Population Health, University of Melbourne, Carlton, VIC 3053, Australia; 3 Department of Colorectal Medicine and Genetics, The Royal Melbourne Hospital, Parkville, VIC 3050, Australia; 4 University of Queensland, Herston, Q4006, Australia; 5 Envoi Pathology, Herston, Q4006, Australia; 6 Familial GI Cancer Registry, Auckland, New Zealand; 7 Department of Gastroenterology, Middlemore Hospital, Auckland, New Zealand

## Background

Smoking has consistently been associated with the development of polyps in the colorectum. However the association between smoking and cancer of the colorectum is less than consistent, a phenomenon known as “the smoking paradox”. Recently, it has been demonstrated that the link between smoking and polyps is strongest in hyperplastic polyps, and in the subset of cancers which develop from hyperplastic polyps. Therefore, given that hyperplastic polyposis is associated with an increased risk for developing colorectal cancer (CRC), the aim of this work was to investigate the association between smoking and the risk of CRC in these high-risk patients.

## Methods

One hundred and fifty-one Caucasian individuals with multiple hyperplastic polyps including at least 5 polyps outside the rectum, were classified into non-smokers, current or former smokers at the time of initial diagnosis of polyposis. Cases were individuals with multiple serrated polyps who presented with CRC. Controls were individuals with multiple serrated polyps and no CRC. Multivariate logistic regression was performed to estimate associations between smoking and CRC with adjustment for age at first presentation, sex, and co-existing traditional adenomas, a feature which has been consistently linked with CRC risk in patients with hyperplastic polyposis.

## Results

CRC was diagnosed in 56 (37%) individuals at initial presentation. Patients with at least one adenoma were 4 times more likely to present with CRC compared with patients without adenomas (OR=4.09; 95%CI 1.27 to 13.14; *P*=0.02). For females, the odds of CRC decreased by 90% in current smokers as compared to never smokers (OR=0.10; 95%CI 0.02 to 0.47; *P*=0.004) after adjusting for age and adenomas. The 2 currently smoking females who presented with CRC both had their polyps concentrated in the recto-sigmoid and had >100 hyperplastic polyps in total. For males, there was no relationship between current smoking and CRC. There was no statistical evidence of an association between former smoking and CRC for both sexes. A decreased odds for CRC was identified in females with multiple serrated polyps who currently smoke, independent of age and the presence of a traditional adenoma. This observation could be seen as a parallel to the observations regarding current smoking and disease in ulcerative colitis, and perhaps suggests that females with hyperplastic polyposis favoring the proximal colon may have an inflammatory basis for their disease.

## Conclusion

Investigations into the biological basis of these observations could lead to non-smoking-related therapies being developed to decrease the risk of CRC and colectomy in patients with hyperplastic polyposis.

